# Microbiological quality assessment of *Clarias gariepinus*, *Bagrus bajad*, and *Pangasianodon hypophthalmus* fillets

**DOI:** 10.1038/s41598-024-62730-8

**Published:** 2024-06-10

**Authors:** Noha M. El-Gendy, Amr Amer, Hossam A. Ibrahim, Mahmoud Abou-Okada

**Affiliations:** 1 Department of Food Hygiene, Faculty of Veterinary Medicine, Alexandria University, Alexandria, 21526 Egypt; 2 Department of Aquatic Animal Medicine and Management, Faculty of Veterinary Medicine, Cairo University, Giza, 12211 Egypt

**Keywords:** Microbiology, Molecular biology, Ichthyology, Environmental impact, Microbiology techniques, DNA sequencing

## Abstract

In this study, 80 catfish fillets were randomly collected from Egyptian local markets and retailers. The samples included 20 African catfish (*Clarias gariepinus)*, 20 bayad (*Bagrus bajad)*, and 40 pangasius catfish (*Pangasianodon hypophthalmus*) fillets. *Pangasianodon hypophthalmus* fillet samples were divided into 20 white basa and 20 red basa fillets. We conducted a microbiological analysis of catfish fillet samples, evaluating mesophilic aerobic bacteria, psychrophilic aerobic bacteria, H_2_S-producing bacteria, *Staphylococcus* spp., *Enterobacteriaceae*, Coliforms, and fecal Coliform counts. Additionally, we identified the existence of *Salmonella* spp., *Vibrio spp*., *Yersinia* spp., *Escherichia* spp., *Aeromonas* spp., and *Pseudomonas* spp. in the catfish fillet samples. In our study, the psychrophilic bacterial counts in *Bagrus bajad* (5.21 log CFU/g) were found to be higher compared to the counts in *Clarias gariepinus* (4.31 log CFU/g) and *Pangasianodon hypophthalmus* (3.89–4.7 log CFU/g). The fecal Coliform in *Clarias gariepinus* fillets was significantly higher than in other catfish fillets. We isolated *Escherichia coli*, *Escherichia fergusonii*, *Aeromonas hydrophila*, and *Pseudomonas luteola* from the catfish fillets, while no *Salmonella* spp., *Vibrio* spp., or *Yersinia* spp. were detected. These isolates were identified using 16S rRNA sequencing and phylogenetic analysis. Furthermore, ten *Escherichia* spp. were serologically identified, revealing that O26 and O78 were the most commonly occurring serotypes. This study highlights the microbiological analysis conducted on catfish fillets and concludes that the fillet samples from these catfish were of superior quality and deemed acceptable for human consumption.

## Introduction

Fish and shellfish possess a high degree of perishability and are susceptible to substantial quality differences attributed to species distinctions, environmental habitats, feeding behaviors, and the influence of both autolysis enzymes and hydrolytic enzymes, produced by microorganisms that affect the fish's muscle tissue^1^.

Catfish are a diverse clade of more than 4100 ray-finned fish species, representing more than 12% of all teleosts, and around 6.3% of all vertebrates^2^. Catfish have been commonly captured and cultured for hundreds of years in Africa, Asia, South America, North America and Europe^3^. African catfish (*Clarias gariepinus*; Burchell, 1822) is an important commercial finfish species in fisheries and aquaculture sectors^4^. African catfish is able to survive and grow in poorly oxygenated water, high stocking densities, grow at a faster rate, resist diseases and handling stress and produce good tasting flesh^5^. Bayad, scientifically known as *Bagrus bajad* (Forsskal, 1775), represents a particular type of bagrid catfish that is observed in a wide range of natural habitats in African lakes and rivers^6^. Pangasius catfish (*Pangasianodon hypophthalmus;* Sauvage, 1878) are one of the most popular riverine freshwater species whose range is limited to the Mekong River and Mekong basins and a yearly turnover of 1.525 million metric tons, are produced in Vietnam^7,8^. Pangasius catfish fillet has been exported to over 138 countries with a value of about 1.6 billion USD^8^.

Fish meat serves as a primary and affordable source of animal proteins, emphasizing and underscoring the significance of proper processing and preservation techniques. Among the various fish species, catfish stand out as particularly suitable for additional utilization due to their remarkable resilience to diverse environmental conditions and their rapid growth rate^9^. A fillet is the edible portion of fish, after removal of the head, fins, viscera, bones, skin, and adipose tissues. The yield of catfish fillets can vary depending on several factors such as the size of the fish, the trimming and cleaning process, and any losses during processing. However, as a general guideline, the yield of catfish fillets is typically around 40–50% of the whole fish’s weight^10^.

Freshness is the most important attribute when assessing the quality of fish. Sensory, microbial, chemical, and physical determination methods can be used to assess fish quality by measuring the lipid oxidation, volatile compounds, TVC (total viable count) and any changes in the sensory attributes of the fish^11–13^. Psychrophilic aerobic bacteria (PAC) are prevalent in numerous environmental settings, and as a result, fish could have acquired them from various pathways including water, harvesting, transportation, handling, processing, distribution and storage^14^. Psychrophilic bacteria are Gram-negative microorganisms found within the genera *Aeromonas*, *Vibrio*, *Acinetobacter*, *Pseudomonas*, *Flavobacterium*, *Photobacterium*, *Shewanella*, and *Moritella* as well as Gram-positive microorganisms found within the genera *Bacillus*, *Arthrobacter, Micrococcus* and *Lactobacillus*^14,15^. The total viable count represents the conventional microbiological technique employed for assessing the quality of finfish fillets and stands as a prevalent quality metric endorsed by food safety authorities^16^. The enumeration of specific spoilage organisms (SSOs) on iron agar provides a more reliable microbial indicator of fish freshness. SSOs play a key role in the spoilage of fish and seafood. While initially present in limited numbers, they proliferate more rapidly than other bacteria within the fish^17,18^.

Foodborne pathogens including *Salmonella* spp., *Yersinia* spp., *Aeromonas* spp., *Pseudomonas* spp., *Staphylococcus aureus*, *Vibrio* spp., and *Escherichia*, play a crucial role in fish-related concerns^19^. These pathogens are responsible for causing foodborne illnesses such as typhoid fever, gastroenteritis, diarrhea, and dysentery. These illnesses pose significant health hazards to consumers, including the risk of death^20^. Fish fillets contamination by *Escherichia* spp. is mainly associated with contaminated water or through cross-contamination during washing, filleting, and trimming of the fish^21^. The spoilage of fresh fish and its products is predominantly attributed to *Aeromonas* spp.^22^. *Pseudomonas* spp. are opportunistic bacteria that thrive in various environments and can be present in varying quantities^22^. This group of microorganisms are commonly encountered in fish and other fresh foods, where they play a role in the spoilage process^23^. *Pseudomonas luteola* is Gram-negative non-spore forming bacilli, catalase-positive, urease negative, indole negative, cytochrome oxidase-negative, H_2_S production negative and oxidation fermentation negative. *Pseudomonas luteola* commonly found in aqueous environments, soil and plants^24,25^.

Catfish fillets are highly favored by consumers because of their nutritional value and favorable sensory characteristics. However, while there is extensive literature on the quality of catfish fillets, there is limited research on the quantitative and qualitative microbiological aspects. Hence, this study was aimed to evaluate the levels of spoilage and pathogenic microorganisms in fillet samples of African catfish (*Clarias gariepinus*), bayad (*Bagrus bajad*), and pangasius catfish (*Pangasianodon hypophthalmus*).

## Methods

### Ethics declarations

The experimental protocols and all methods were performed in accordance with the ARRIVE 2.0 standards (Animal Research: Reporting of In Vivo Experiments) guidelines and regulations. All procedures and protocols for experiments were carried out in compliance with the guidelines and regulations set by Veterinary Medicine Cairo University Institutional Animal Care and Use Committee (Vet. CU. IACUC; Vet CU18042024918).

### Sample size calculation

The determination of the sample size for this study followed the formula for an unknown population, as outlined by Kothari^26^: n = Z^2^SD^2^/e^2^, In this calculation, where Z represents the value of the standard variate (1.96) at a 95% confidence level, SD denotes the standard deviation of the population derived from the trial sample (0.11), and e stands for the tolerable sampling error or precision (0.05) within a 95% confidence interval. Subsequently, the sample size was computed as:

$$\text{n}=\frac{{Z}^{2}{SD}^{2}}{{e}^{2}}\text{ n}=\frac{{(1.96)}^{2}{(0.11)}^{2}}{{(0.05)}^{2}} \approx 19\; \text{samples }$$

Therefore, the sample size of each catfish fillet type was 19, making a total of 80 samples for the four types of fillets (African catfish fillets, Bayad fillets, white basa fillets and red basa fillets).

### Samples collection

In total, 80 samples were collected. This included 20 African catfish (*C. gariepinus*) fillets weighing between 600 and 1000 g, 20 bayad (*B. bajad*) fillets weighing between 500 and 2500 g and 40 skinless-frozen pangasius catfish (*P. hypophthalmus*) fillets weighing between 600 and 800 g. These samples were obtained from fish markets and retailers in Egypt (Kafr El Sheikh, Alexandria and Beheira governorates) between 2021 and 2023. Pangasius catfish fillets on the Egyptian market can be divided into two grades: white basa fillets (20 samples) and red basa fillets (20 samples). All catfish fillet samples were transported in separate iceboxes filled with ice bags to the Department of Food Hygiene, Alexandria University. Microbiological analyses were performed immediately on the catfish fillet samples.

### Microbiological analyses

#### Enumeration of bacterial load

Upon arrival at the laboratory, sterile scalpels and tweezers were used to aseptically collect 25 g samples of fish fillet, which were then placed in a sterile stomacher bag. Next, 225 mL of sterile 0.1% peptone water (Difco, UK) was added to the bag, and the mixture was homogenized for 1 min using a Stomacher at the normal speed (Stomacher lab-blender 400, Seward Medical, UK) according to ISO 6887–3:2017^27^. Subsequently, a tenfold serial dilution series was prepared, and the counts were determined using the pour plate technique according to ISO 6887–3:2017^27^. Each analysis was conducted twice in order to ensure accuracy. The number of viable microorganisms was then counted, calculated, and expressed as the logarithm of colony-forming units per gram (log CFU/g).

Plate Count Agar (PCA) is a widely employed solid culture medium for enumeration the viable bacterial population in a sample. It provides a nutrient-rich environment that supports the growth of a wide range of bacteria. PCA contains a combination of peptones, yeast extract, and agar according to ISO 4833–1:2013^28^. The mesophilic aerobic count and psychrophilic aerobic bacteria were determined using a Plate Count Agar (PCA, Oxoid), then incubated at 30 °C for 48 h and at 7 °C for 7–10 days, respectively according to according to ISO 6887–3:2017^27^**;** ISO 4833–1:2013^28^. The hydrogen sulfide producing bacteria were enumerated using iron agar (14 g agar, 3 g beef extract, 20 g peptone, 5 g sodium chloride, 3 g yeast extract, 0.6 g L-Cysteine, 0.3 g Sodium thiosulfate and 0.3 g Ferric citrate per 1L autoclaved distilled water) according to Gram et al*.*^29^. *Staphylococcus* spp. and *Staphylococcus aureus* counts using Baird-Parker agar medium according to ISO 6888:2021^30^. *Enterobacteriaceae* counts were enumerated using Violet red bile glucose (VRBG) agar and incubated at 37 °C for 24 h according to ISO 21,528:2017^31^. The Most Probable Number (MPN) method was used for the enumeration of Coliform and fecal Coliform according to Feng et al*.*^32^**;** ISO 7251:2005^33^; ISO 4831:2006 ^34^; Oblinger & Koburger^35^.

#### Isolation of pathogenic *bacteria*

Isolation of *E. coli* was conducted according to ISO 16,649:2018^36^. Approximately 1 g of homogenized fish fillets was mixed with 9 mL of modified Tryptone Soya Broth (mTSB, HiMedia). The samples were thoroughly mixed and left to incubate overnight at a temperature of 41 °C. Following selective enrichment, 50 µL of the resulting mixture were spread onto MacConkey agar (HiMedia) plates to isolate *E. coli* bacteria, and the plates were incubated aerobically at 37 °C for 24 h. The plates were then examined for the presence of *E. coli* growth, characterized by pink colonies indicating lactose fermentation. A single, isolated colony exhibiting these characteristics was chosen and transferred to Eosin Methylene Blue agar (EMB, HiMedia) to observe the formation of a metallic sheen. At the same time, another colony displaying similar characteristics was subjected to gram staining according to ISO 16,649:2018^36^.

Isolation of *Salmonella* spp. was performed according to ISO 6579:2017^37^. Briefly, 225 mL of buffered peptone water was inoculated with 25 g fish fillets and incubated at 37 °C for 18 h (pre-enrichment in non-selective liquid medium), then inoculate 1 mL of the above-mentioned broth into 9 mL Rappaport–Vassiliadis medium with soya (RVS broth, Oxoid) and finally plating out on Xylose Lysine Deoxycholate agar (XLD, Oxoid) and Salmonella Shigella agar (SS, Oxoid), incubated at 37 °C for 24 h according to ISO 6579:2017^37^.

*Yersinia* spp. was performed according to ISO 10,273:2017^38^. Isolation of *Yersinia* spp. was starting from enrichment in non-selective PSB broth (HiMedia), and then direct plating from PSB broth onto Cefsulodin-Irgasan-Novobiocin (CIN, HiMedia) agar plates, incubated at 30 °C for 24 h according to ISO 10,273:2017^38^.

*Vibrio* spp. were performed according to ISO 21,872:2017^39^. Isolation of *Vibrio* spp. was starting from primary enrichment medium alkaline saline peptone water (ASPW, Oxoid), followed by incubation at 37 °C for 6 h, then streaked on thiosulfate citrate bile and sucrose agar (TCBS, Oxoid) and incubated at 37 °C for 24 h according to ISO 21,872:2017^39^.

Representative colonies were selected from the plate count agar (PCA, Oxoid) after incubation at 7°C for 7–10 days. Selected colonies were streaked onto Rimler-Shotts (RS, HiMedia) agar supplemented with Novobiocin (HiMedia), and *Pseudomonas* agar base enriched with Cetrimide, Fucidin, and Cephalosporin (*Pseudomonas* CFC agar, Oxoid) and then placed in an incubator at 25 °C for a period of 24 to 72 h. Typical *Aeromonas* spp. produce greenish-yellow to yellow colonies, or yellow colonies with black centers (H_2_S producing bacteria) on RS agar, while typical *Pseudomonas* spp. produce blue-green colonies on *Pseudomonas* CFC agar.

#### Phenotypic characterization of isolates

The presumptive identification of isolates was accomplished by assessing their phenotypical characteristics according to the criteria described by Bergey^40^. Biochemical characterization of the isolates using commercial miniaturized API-20E (Biomérieux, France) were performed according to the manufacturer’s instructions. Confirmed isolates were maintained until further use at − 20°C in nutrient broth containing 16% glycerol.

#### Serological identification of *Escherichia* spp. isolates

A total of ten isolates of *E. coli* and *E. fergusonii*, which were identified based on their phenotypic characteristics, underwent serological identification according to Ewing^41^.

#### Genotypic characterization, 16S rRNA sequencing and Phylogenetic analysis

Four bacterial isolates were selected for sequencing studies based on their morphological and biochemical characteristics. The selected bacterial isolates were *Aeromonas hydrophila,* isolated from *Bagrus bajad*, *Pseudomonas luteola,* isolated from *Pangasianodon hypophthalmus* as well as *E. coli*, and *E. fergusonii,* isolated from *Clarias gariepinus*. The DNA extraction of these isolates was performed using QIAamp DNA Kits (Qiagen, USA) following the instructions provided by the manufacturer. The DNA was maintained until further use at − 20 °C. The genotypic identification of isolates was confirmed by employing universal 16S rRNA gene primers (Forward: 5′-AGAGTTTGATCCTGGCTCAG-3′) (Reverse: 5′-GGTTACCTTGTTACGACTT-3′)^42^. The 16S rRNA gene was amplified using 50 µL reaction volume in Maxima Hot Start PCR Master Mix (ThermoFisher, USA), per the manufacturer’s instructions. The PCR procedure was set with an initial denaturation step at 95 °C for 10 min, followed by 35 cycles of denaturation at 95 °C for 30 s, annealing at 65 °C for 60 s, and extension at 72 °C for 90 s. The final extension step was conducted at 72 °C for 10 min. The PCR products were examined using a 1% (w/v) agarose gel that was treated with ethidium bromide for staining^42^.

The sequences were examined using an ABI 3730xl DNA sequencer from Applied Biosystems™ in the Sigma Scientific Services Laboratory located in Cairo, Egypt. The 16S rRNA sequences acquired were then compared to existing databases through BLASTN on NCBI to determine their closest phylogenetic affiliations^43^. The neighbor-joining algorithm in MEGA X was utilized to construct the phylogenetic analysis^44^. The evolutionary history of the analyzed taxa was represented by constructing a bootstrap consensus tree, which was generated from 500 replicates. The Maximum Composite Likelihood method, developed by Tamura et al*.*^45^, was used to calculate evolutionary distances. These distances are expressed as the number of base substitutions per site^46^.

### Statistical analyses

Statistical analyses were performed via the R program (R 4.3.1)^47^. The heteroscedasticity of variances and the normality of residuals was calculated using the Levene's and Shapiro–Wilk tests. Microbiological assessment data of catfish fillets was presented as the mean ± *SEM* (Standard error of mean). Microbiological data were computed by one way analysis of variance (ANOVA) and followed by Tukey’s post hoc test for multiple comparison between groups. The significance level was set at a probability value of less than 0.05 (*p* ˂ 0.05).

## Results

### Quantitative microbiological analyses

Mesophilic aerobic count, psychrophilic aerobic bacteria, H_2_S producing bacteria, *Staphylococcus* spp., *Staphylococcus aureus, Enterobacteriaceae*, Coliform and fecal Coliform counts for each type of fillet are shown in Table 1. The mesophilic aerobic count, *Staphylococcus* spp., *Staphylococcus aureus*, hydrogen sulfide producing bacteria, *Enterobacteriaceae* and Coliform counts displayed no significant differences (*p* > 0.05) among the examined samples. Psychrophilic aerobic bacteria of bayad fillets (> 5 log CFU g^−1^) were significantly higher bacterial counts than African catfish fillets (> 4 log CFU g^−1^) and pangasius catfish fillets (white basa fillets only: ≤ 4 log CFU g^−1^). Although *Staphylococcus* spp. count in pangasius catfish fillet samples were higher than 4 log CFU g^-1^, *Staphylococcus aureus* was estimated to be 1.33 to 1.51 log CFU g^-1^ (Table 1). Hydrogen sulfide producing bacterial counts in pangasius catfish fillets (red basa fillets; 2.91 log CFU g^−1^) revealed higher H_2_S bacterial counts than African catfish fillets (2.65 log CFU g^−1^), bayad fillets (2.33 log CFU g^−1^) and pangasius catfish fillets (white basa fillets; 2.57 log CFU g^−1^). The *Enterobacteriaceae* count in pangasius catfish fillets samples was 2.86 to 3.01 log CFU g^-1^ for white basa and red basa. African catfish fillet (1.27 log MPN g^−1^) showed significantly higher fecal Coliform counts than bayad fillets (0.91 log MPN g^−1^) and Pangasius catfish fillets (0.50–0.56 log MPN g^−1^). The fecal Coliform counts are shown in Table 1.

**Table 1:** Bacterial load of examined African catfish (*Clarias gariepinus*), bayad (*Bagrus bajad*) and pangasius catfish (*Pangasianodon hypophthalmus*) fillets.

Bacterial load	African catfish	Bayad	White basa	Red basa
Mesophilic aerobic count (log CFU g^-1^)	4.96^a^ ± 0.117	4.72^a^ ± 0.145	4.82^a^ ± 0.275	5.07^a^ ± 0.136
Psychrophilic aerobic count (log CFU g^-1^)	4.31^b^ ± 0.201	5.21^a^ ± 0.287	3.89^b^ ± 0.212	4.70^ab^ ± 0.140
*Staphylococcus* spp. count (log CFU g^-1^)	3.79^a^ ± 0.374	3.71^a^ ± 0.362	4.18^a^ ± 0.302	4.39^a^ ± 0.188
*Staphylococcus aureus* count (log CFU g^-1^)	1.58^a^ ± 0.154	1.40^a^ ± 0.175	1.33^a^ ± 0.129	1.51^a^ ± 0.192
H_2_S producing bacteria count (log CFU g^-1^)	2.65^a^ ± 0.204 (7)	2.33^a^ ± 0.095 (4)	2.57^a^ ± 0.061 (2)	2.91^a^ ± 0.124 (4)
*Enterobacteriaceae* count (log CFU g^-1^)	3.42^a^ ± 0.181	3.15^a^ ± 0.209	2.86^a^ ± 0.205	3.01^a^ ± 0.101
Coliform count (log MPN g^-1^)	2.40^a^ ± 0.135	2.28^a^ ± 0.132	2.03^a^ ± 0.092	2.19^a^ ± 0.091
Fecal Coliform count (Log MPN g^-1^)	1.27^a^ ± 0.044 (5)	0.91^b^ ± 0.109 (3)	0.50^c^ ± 0.027 (2)	0.56^c^ ± 0.031 (2)

Data represented as mean of 20 samples (duplicate) ± *SEM*- Standard error of the mean, rows with distinct superscripts exhibited statistically significant differences, as determined by ANOVA and Tukey's post-hoc test, with a significance level of *p* ≤ 0.05. Pangasius catfish fillets can be divided into two grades: white basa fillets (grade I, white colour) and red basa fillets (grade II, pale pink to red colour). The number between parenthesis refers to number of positive samples out of 20 samples.

### Qualitative microbiological analyses

In the present study, concerning *Salmonella* spp., *Yersinia* spp. and *Vibrio* spp., no colony was isolated from our catfish fillet samples. On the other hand, *Escherichia* spp. was detected in five African catfish fillet samples, three bayad fillet samples and two pangasius catfish fillet samples (Table 2). The incidence of *Escherichia* spp. isolated from catfish fillets were 25%, 15%, 5% and 5% for African catfish fillet samples, bayad fillet samples and white basa fillet samples and red basa fillet samples. In the other hand, the incidence of *Aeromonas* spp. isolated from catfish fillets were 25%, 20%, 5% and 20% for African catfish fillet samples, bayad fillet samples, white basa fillet samples and red basa fillet samples. Additionally, the incidence of *Pseudomonas* spp. isolated from pangasius catfish fillets were 5% and 15% for white basa fillet samples and red basa fillet samples (Table 2).

**Table 2:** Pathogenic bacterial isolates retrieved from African catfish (*Clarias gariepinus*), bayad (*Bagrus bajad*) and pangasius catfish (*Pangasianodon hypophthalmus*) fillets.

Microorganism	African catfish	bayad	White basa	Red basa
*Escherichia* spp.	5 (25%)	3 (15%)	1 (5%)	1 (5%)
*Salmonella* spp.	ND	ND	ND	ND
*Yersinia* spp.	ND	ND	ND	ND
*Vibrio spp.*	ND	ND	ND	ND
*Aeromonas* spp.	5 (25%)	4 (20%)	1 (5%)	4 (20%)
*Pseudomonas* spp.	ND	ND	1 (5%)	3 (15%)

*ND*- Not detected. Pangasius catfish fillets can be divided into two grades: white basa fillets (grade I, white colour) and red basa fillets (grade II, pale pink to red colour).

### Phenotypic and genotypic characterization of isolates

**Figure 1:**
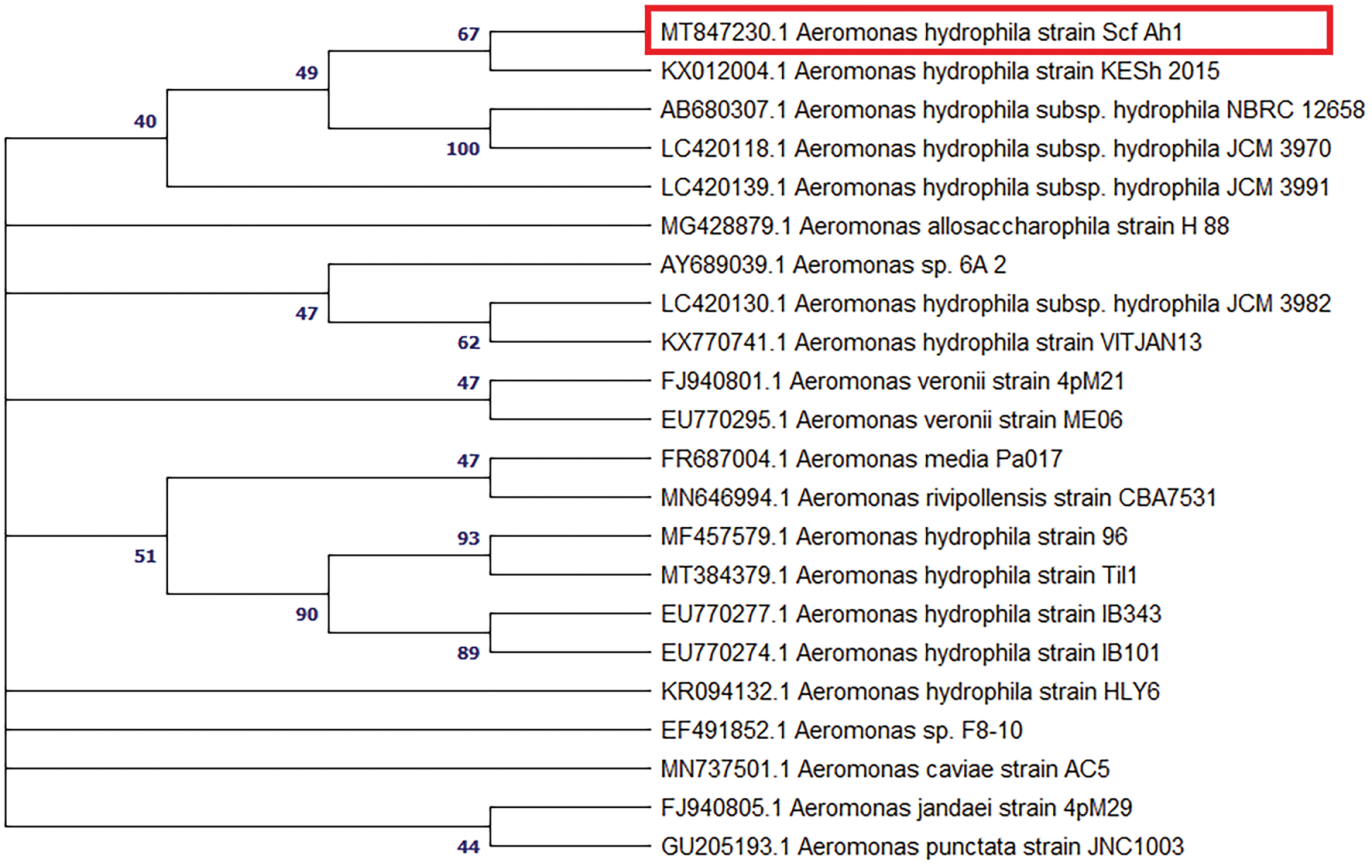
Presents a phylogenetic tree of twenty identified *Aeromonas* spp. and *Aeromonas hydrophila* (Scf Ah1) isolate *(Bagrus bajad)*, represented by a red rectangle. The tree was constructed using the neighbor-joining method, and the aligned sequence had a length of 1485 bp. The bootstrap values (%) are displayed next to the clades, and the accession numbers are indicated before the strain names. The neighbor-joining tree was constructed using MEGA X, scale 0.05.

**Figure 2:**
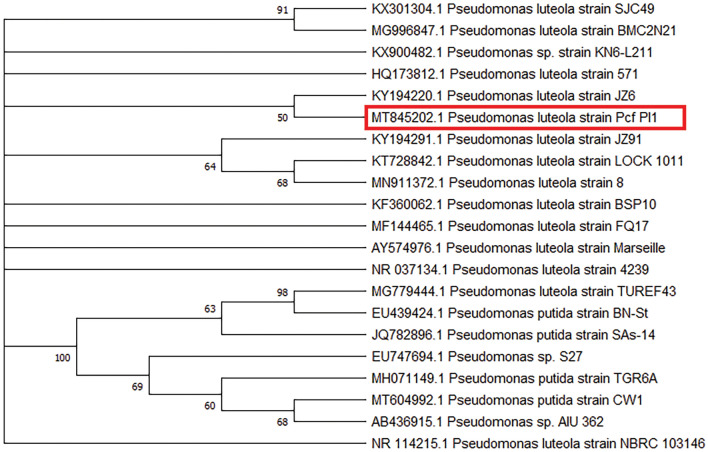
Presents a phylogenetic tree of twenty identified *Pseudomonas* spp. and *Pseudomonas luteola* (Pcf Pl1) isolate (*Pangasianodon hypophthalmus*), represented by a red rectangle. The tree was constructed using the neighbor-joining method, and the aligned sequence had a length of 1490 bp. The bootstrap values (%) are displayed next to the clades, and the accession numbers are indicated before the strain names. The neighbor-joining tree was constructed using MEGA X, scale 0.05.

The results of the phenotypic analyses conducted on *P. luteola* and *A. hydrophila* in the current study are presented in Table 3. The pure cultures of *A. hydrophila* and *P. luteola* were confirmed by sequencing 16S rRNA genes. Compared to the 1485 bp 16S rRNA gene of *A. hydrophila* (MT847230) expressed a 99.5% homology with the 16S rRNA sequence of *A. hydrophila subsp. hydrophila* (LC420139, KX012004 and LC420130)**,** whereas the sequences available in the GenBank, 1490 bp 16S rRNA gene of *P. luteola* (MT845202) expressed a 99.6% homology with the 16S rRNA sequence of *P. luteola* (KT728842, KY194220 and KY194291). The phylogenetic tree using 16S rRNA gene sequences of *A. hydrophila* and *P. luteola* is shown in Figs. 1 and 2, respectively.

**Table 3:** Phenotypic characterization of isolates retrieved from African catfish (*Clarias gariepinus*), bayad (*Bagrus bajad*) and pangasius catfish (*Pangasianodon hypophthalmus*) fillets.

	*Escherichia coli* (n= 8)	*Escherichia* *fergusonii* (n= 2)	*Aeromonas hydrophila* (n= 14)	*Pseudomonas luteola* (n= 4)
Gram stain	*Gram-ve, rod shaped*	*Gram-ve, rod shaped*	*Gr-ve, rod shaped*	*Gr-ve, rod shaped*
Motility	*+*	*+*	*+*	*+*
Conventional tube media				
Oxidase	–	–	+	–
Catalase	+	+	+	+
Indole	+	+	+	–
Citrate utilization	–	–	+	+
Urea hydrolysis	–	–	–	–
Triple Sugar Iron (TSI)	A/Ag	A/Ag	K/A	K/K
Methyl Red (MR)	+	+	+	–
Voges Proskauer (VP)	–	–	+	–/+
Oxidation/fermentation	O/F	O/F	O/F	O
API-20E				
ONPG (ß-galactosidase)	+	–	+	+
ADH (Arginine DiHydrolase)	+/–	+	+	+
LDC (Lysine DeCarboxylase)	+	+	–	–
ODC (Ornithine DeCarboxylase)	+	+	–	–
CIT (Citrate utilization)	–	–	+	+
H_2_S (H_2_S production)	–	–	–	–
URE (Urease)	–	–	–	–
TDA (Tryptophane DeAminase)	–	–	–	–
IND (Indole production)	+	+	+	–
VP (Voges Proskauer)	–	–	–	–/+
GEL (Gelatinase)	–	–	+	–
GLU (Glucose)	+	+	+	+
MAN (Mannitol)	+/–	+	+	–
INO (Inositol)	–	–	–	–
SOR (Sorbitol)	+/–	–	+	–
RHA (Rhamnose)	+	+	–	–
SAC (Saccharose)	–/+	–	+	–
MEL (Melibiose)	+/–	–	–	+
AMY (Amygdalin)	–	+	+/–	–
ARA (Arabinose)	+/–	+	+	+
OX (Cytochrome Oxidase)	–	–	+	–

+ : Positive, –: Negative, –/ + : Most isolates are negative, + /–: Most isolates are positive, A/Ag: Acid slant (yellow) and acid butt (yellow) with gases, K/A: Alkaline slant (red) and acid butt (yellow), K/K: No change in slant (red) and alkaline butt (red). O/F: Oxidation fermentation positive, O: Oxidation positive. Number in parenthesis indicated number of isolates identified by phenotypic characterization.

**Figure 3:**
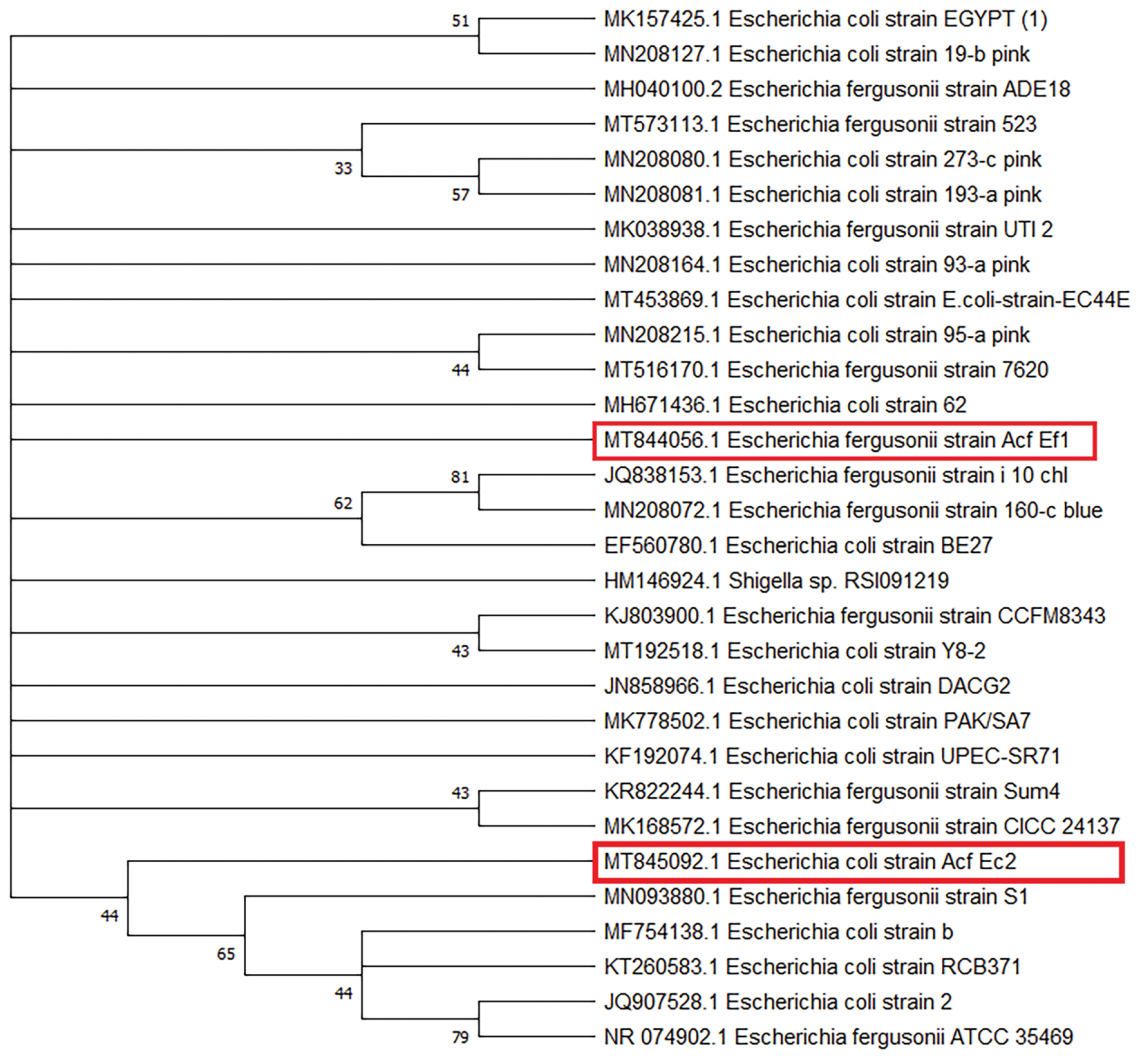
Presents a phylogenetic tree of twenty-eight identified *Escherichia* spp. and *E. coli* (Acf Ec2), and *E. fergusonii* (Acf Ef1) isolates (*Clarias gariepinus*), represented by red rectangles. The tree was constructed using the neighbor-joining method, with the aligned sequences having lengths of 1432 bp and 1386 bp, respectively. The bootstrap values (%) are displayed next to the clades, and the accession numbers are indicated before the strain names. The neighbor-joining tree was constructed using MEGA X, scale 0.05.

The results of the phenotypic analyses conducted on *E. coli* and *E. fergusonii* in the current study are presented in Table 3. The serological identification of eight *E. coli* and two *E. fergusonii* isolates are shown in Table 4. In this study, we isolated *E. coli* serotype O26 from African catfish, bayad, and pangasius catfish fillets, while *E. fergusonii* serotype O78 was found in two African catfish fillet samples. Following 16S rRNA sequencing analysis, the phenotypically and serologically identified *Escherichia* isolates were assigned to *E. coli* and *E. fergusonii*. Additionally, the construction of the phylogenetic tree utilizes 16S rRNA gene sequences, with length of 1432 bp (*E. coli*, MT845092) and 1386 bp (*E. fergusonii*, MT844056) is shown in Fig. 3. Compared to the sequences available in GenBank, *E. fergusonii* 16S rRNA gene (MT844056) expressed a 99.5% homology with the 16S rRNA sequence of *E. fergusonii* (JQ838153 and MH040100), while *E. coli* 16S rRNA gene (MT845092) expressed 99.9% homology with the 16S rRNA sequence of *E. coli* (KT260583 and MF754138). This study reports the first isolation of *E. fergusonii* from African catfish fillet samples.

**Table 4:** Serotyping and API-20E code of *Escherichia* spp. isolates (n = 10).

Isolate code	Fish species	*Escherichia* spp.	API-20E code	Serogroups
Acf_Ec1	African catfish (*C. gariepinus*)	*E. coli*	5144110	O127
Acf_Ec2		*E. coli*	5144052	O26
Acf_Ec3		*E. coli*	7144512	O126
Acf_Ef1		*E. fergusonii*	6144113	O78
Acf_Ef2		*E. fergusonii*	6144113	O78
Scf_Ec1	bayad (*B. bajad*)	*E. coli*	5144552	O6
Scf_Ec2		*E. coli*	7144572	O26
Scf_Ec3		*E. coli*	5144532	O128
Pcf_Ec1	Pangasius catfish *(P. hypophthalmus)*	*E. coli*	5144052	O26
Pcf_Ec2		*E. coli*	7144012	O86

## Discussion

In this study, we evaluate the levels of spoilage and pathogenic microorganisms in catfish fillet samples. Most of the evaluated microbiological properties are within the permissible limits set by International Commission on Microbiological Specification for Food^48^. Psychrophilic aerobic bacteria of bayad fillets (> 5 log CFU g^−1^) were significantly higher than African catfish fillets (> 4 log CFU g^−1^) and pangasius catfish fillets (white basa fillets only: ≤ 4 log CFU g^−1^). Dambrosio et al*.*^14^ indicated that the average psychrophilic aerobic bacteria count in fillet samples of *P. hypophthalmus*, acquired from an Italian trade import services company, was 4.44 log CFU g^-1^ and these results were comparable with our results. Moreover, the counts of aerobic psychrotrophic microorganisms found in pangasius catfish varied from 4.6 to 5.9 log CFU g^-1^^21^. Nevertheless, although CFU g^-1^ obtained in this study was moderately high, it did not exceed the acceptable permissible limit for total bacterial load (5.5–7.0 log CFU g^-1^) for fresh and frozen fish, as established by International Commission on Microbiological Specification for Food^48^. The elevated levels of psychrophilic aerobic bacteria found in bayad fillets may be attributed to the preservation method commonly used for bayad in Egyptian markets, which involves storing it with an equal amount of crushed ice.

*Staphylococcus aureus* does not naturally inhabit the microbiota of fish. Consequently, its occurrence in fish is possibly linked to unsanitary practices during handling by fish handlers, processors, or sellers, as well as potential cross-contamination throughout handling, transportation, storage, and processing, stemming from the presence of this pathogen in the microbiome of most humans^49–51^. Although *Staphylococcus* spp. count in our pangasius catfish fillet samples were higher than 4 log CFU g^-1^, *Staphylococcus aureus* was estimated to be 1.33 to 1.51 log CFU g^-1^. These moderately high levels suggested that the product contamination is possibly linked to unsanitary practices during handling, processing, selling, and storage etc.^50–52^. Tong Thi et al*.*^21^ indicated that the detection of *Staphylococcus aureus* on the hands of food operators during fish processing, especially in the packaging area, was deemed indicative of inadequate personal hygienic practices. Lower *Staphylococcus aureus* level (1.14 log CFU g^-1^) in pangasius catfish fillets was previously reported by Dambrosio et al*.*^14^. Our findings were lower than the acceptable permissible limit of *Staphylococcus aureus* (˂ 3 log CFU g^-1^) in fish fillets set by Egyptian Organization for Standardization and Quality^53^.

The quantification of specific spoilage organisms (SSOs) on iron agar is a more reliable microbial measure of fish freshness; SSOs are responsible for the deterioration of fish and seafood^17,18^. Hydrogen sulfide producing bacterial counts in pangasius catfish fillets (red basa fillets; 2.91 log CFU g^−1^) revealed higher counts than African catfish fillets (2.65 log CFU g^−1^), bayad fillets (2.33 log CFU g^−1^) and pangasius catfish fillets (white basa fillets; 2.57 log CFU g^−1^). The *Enterobacteriaceae* count in pangasius catfish fillets samples was 2.86 to 3.01 log CFU g^-1^ for white basa and red basa, which is higher than the *Enterobacteriaceae* count in pangasius catfish fillets samples (2.29 log CFU g^-1^) previously reported by Dambrosio et al*.*^14^. *Enterobacteriaceae* and Coliform levels in fish fillets are an indicator of general bacteriological conditions, and an index for the presence of pathogenic enteric organisms^54^. Mossel and Tamminga^55^ adopted a reference value (3 log CFU g^−1^) for *Enterobacteriaceae* in fish fillets. In the present study, the mesophilic aerobic counts, psychrophilic aerobic bacteria, *Staphylococcus aureus* and *Enterobacteriaceae* counts of catfish fillets were within the acceptable permissible limit set by Egyptian Organization for Standardization and Quality^53^**;** International Commission on Microbiological Specification for Food^48^.

Fecal Coliforms are a group of bacteria most commonly used as pollution indicators in food and water and easily affected by freezing storage^56^. The fecal Coliform count in African catfish fillet (1.27 log MPN g^−1^) was significantly higher than bayad fillets (0.91 log MPN g^−1^) and pangasius catfish fillets (0.50–0.56 log MPN g^−1^). These levels were lower than the upper acceptable permissible limits of fecal Coliform for fish fillets set by Egyptian Organization for Standardization and Quality^53^**;** International Commission on Microbiological Specification for Food^48^. Comparable results were previously documented by Budiati et al*.*^57^, who observed that the fecal Coliform content for catfish ranged between 0.48 and 1.63 log MPN g^-1^. The lower levels of fecal Coliform found in white basa (0.50 log MPN g^-1^), and red basa (0.56 log MPN g^-1^) fillets may be attributed to the freezing preservation method commonly used for basa fillets in Egyptian markets. Boyd and Tanner^58^ reported that the high organic matter, poor water quality, inferior feed quality and high stocking density of catfish in ponds could be associated with rising Coliforms and fecal Coliform loads in catfish fillets. Additionally, Budiati et al*.*^57^ suggested that the type of feed can influence the bacterial burden in fish. Utilizing chicken offals and spoiled eggs as fish feed may pose potential sources of bacterial contamination in both fish and aquatic environment.

*Enterobacteriaceae*, Staphylococcus spp., and various other microorganisms may be present in the initial microbial population, primarily as contaminants^59^. No *Salmonella* spp. was detected in our fillet samples and comparable findings were previously documented by Dambrosio et al*.*^14^ who did not detect *Salmonella* spp. from *P. hypophthalmus* fillet samples imported to Italy. Similar findings were reported regarding *Vibrio* spp. by Noseda et al*.*^60^, where they did not detect *Vibrio* spp. in *P. hypophthalmus* fillets. Nevertheless, in contradiction to our results, Tong Thi et al*.*^21^ detected *V. cholerae* (in 1/9 samples) of the pangasius catfish sampled at the filleting step.

*Escherichia* spp. is the predominant Coliform found in the intestinal flora of warm-blooded animals and is primarily linked to fecal contamination^60^. During the processing stage, high levels of *E. coli* were detected in the samples collected from hands and surfaces due to cross contamination from food contact surfaces (hands, cutting boards and knives) and fish fillets^21,60^. Moreover, the presence of *Escherichia* spp. in fish fillet might be attributed to the contamination of fishponds by livestock waste^61^. Yagoub^62^ claimed that the fertilization of the fishpond using farm animal and poultry manure could be a source of *E. coli* in the fish samples. In contrast, low *Escherichia* spp. was detected in pangasius catfish fillets, suggesting that the freezing process had lethal effect on *Escherichia* spp. Fish fillets contamination by *Escherichia* spp. may be associated with contaminated water or through cross-contamination during washing, filleting, and trimming of the fish^21^.

The incidence of *Aeromonas* spp. isolated from catfish fillets were 25%, 20%, 5% and 20% for African catfish fillet samples, bayad fillet samples, white basa fillet samples and red basa fillet samples. According to Henin^63^ & Ibrahim et al*.*^64^**,** the incidence of *Aeromonas* spp. in imported frozen fish, fresh catfish and freshwater fish was reported as 15.2%, 11.6% and 9.7%, respectively. Wong et al*.*^65^ detected *Aeromonas* spp. in 10% of the frozen fish samples they examined. In contrast, *Pseudomonas* spp. was exclusively detected in samples of pangasius catfish fillet. Higher results were reported by Yagoub^62^ who isolated *Pseudomonas* spp. from 62% of the examined fish samples and Rahmou^66^ who isolated *Pseudomonas* spp. from 28% of the examined fish fillet samples. The specific spoilage organisms (SSOs) in the present study were *Aeromonas* spp. and *Pseudomonas* spp., these results agree with Viji et al*.*^67^. Previous studies mostly defined the SSOs in aerobically stored fish and fish products as Gram-negative psychrotrophic bacteria, including *Pseudomonas* spp., *Aeromonas* spp., *Vibrio* spp., and *Shewanella* spp.^18,22,68,69^. Pseudomonads are one of the most significant spoilage organisms, as their rapid growth contributes to the breakdown of nitrogenous compounds, ultimately resulting in the deterioration of product^22^.

*Aeromonas hydrophila* (*A. hydrophila*) is ubiquitous in the aquatic environment and has been found in freshwater fish, including catfish and tilapia^70–72^. *Pseudomonas luteola* are Gram-negative, aerobic, oxidase-negative rods commonly found in aqueous environments, soil and plants^24,25^. *Pseudomonas luteola* is not a common pathogen in aquaculture; the first record of *P. luteola* infection in rainbow trout (*Oncorhynchus mykiss*) was reported by Altinok et al*.*^24^. This study reports the isolation of *P. luteola* in pangasius catfish fillets imported to Egypt.

*E. coli* is ubiquitous, as it naturally inhabits the intestines of warm-blooded animals without causing any symptoms, and it is extensively spread throughout the environment^73^. Thus, *E. coli* is a reliable indicator of fecal contamination, water pollution and mishandling^74,75^. *E. fergusonii* is an emerging opportunistic pathogen and is occasionally isolated from the intestinal contents of human and warm-blooded animals. Several studies have isolated *E. fergusonii* from mammals and birds with systemic or enteric infections^76,77^, whereas a few studies have isolated *E. fergusonii* from sewage, surface water, well water and cultured Egyptian Nile tilapia with signs of bacteremia^78,79^. *E. fergusonii* has been frequently identified in the fecal matter of cattle, poultry, goats, sheep, and horses exhibiting symptoms such as diarrhea, meningitis, mastitis, abortion, and septicemia^76,80^. The phenotypic analysis of *E. fergusonii* were nearly similar to isolates recovered from Egyptian Nile tilapia^78^ except our isolates were positive for ADH (Arginine DiHydrolase) and negative for ONPG (ß-galactosidase).

*E. coli* serotype O26 was frequently isolated from African catfish, bayad, and pangasius catfish fillets, while *E. fergusonii* serotype O78 was found in two African catfish fillet samples. Certain serotypes (O26) found in African catfish were comparable to the predominant *Escherichia* serotypes identified in broiler chickens in Egypt^81,82^. *E. coli* is an extrinsic microorganism for fish environment, and it is not a part of fish flora. *E coli* might be introduced to fishponds through the traditional fertilization of fishponds using farm animals and poultry manure, which may harbor *E. coli*, *E. fergusonii* and other *Enterobacteriaceae* members^21,61,75^.

## Conclusion

The present study aimed to assess the bacterial load and pathogenic bacteria in African catfish, bayad, and pangasius catfish fillets. Our findings indicate that all examined catfish fillets were deemed acceptable and safe for human consumption. No *Salmonella* spp., *Yersinia* spp., or *Vibrio* spp. were detected in any of the examined catfish fillets. *E. coli* serotype O26 was frequently isolated from African catfish, bayad, and pangasius catfish fillets, while *E. fergusonii* serotype O78 was found in two African catfish fillet samples. Furthermore, this study reports first isolation of *E. fergusonii* from African catfish fillets and *Pseudomonas luteola* from pangasius catfish fillets. The isolation of *E. fergusonii* from African catfish fillet samples highlights the need for more research on emerging pathogens and their prevalence in catfish production. To prevent contamination, recontamination, or the survival of biological hazards during handling, processing, distribution, and storage of catfish fillets, we highly recommend implementing Good Manufacturing Practices (GMP), Good Hygiene Practices (GHP), and a meticulously planned HACCP program. Continued surveillance and investigation of bacterial species can contribute to better understanding and management of risks associated with catfish fillets. Overall, the catfish industry, producers and consumers will benefit from using our data on microbiological quality assessment of the catfish fillets for stringent process control.

## Data Availability

The datasets analysed during the current study are available in the GenBank database under the accession numbers: MT844056, MT845092, MT845202 and MT847230.
